# The effect of cycling on cognitive function and well-being in older adults

**DOI:** 10.1371/journal.pone.0211779

**Published:** 2019-02-20

**Authors:** Louise-Ann Leyland, Ben Spencer, Nick Beale, Tim Jones, Carien M. van Reekum

**Affiliations:** 1 School of Psychology and Clinical Language Sciences, University of Reading, Reading, United Kingdom; 2 Dementia Research Centre, Institute of Neurology, University College London, London, United Kingdom; 3 School of the Built Environment, Oxford Brookes University, Oxford, United Kingdom; 4 Centre for Movement, Occupational and Rehabilitation Sciences (MOReS), Oxford Brookes University, Oxford, United Kingdom; University of Rome, ITALY

## Abstract

It has been demonstrated that, on their own, both exercise and stimulation from the environment can improve cognitive function and well-being in older adults. The combined effect of exercising in the outdoor environment on psychological function is less well studied. The aim of the current study was to investigate the effect of an outdoor cycling intervention on cognitive function and mental health and well-being in older adults. A total of 100 older adults took part in the study (aged 50–83), 26 of which were non-cycling controls, 36 were conventional pedal cyclists and 38 were participants using an e-bike (a bike fitted with an electric motor to provide assistance with pedaling), as part of a larger project (www.cycleboom.org). Participants took part in the study for an eight-week period, with cycling participants required to cycle at least three times a week for thirty minutes in duration for each cycle ride. Cognitive function and well-being were measured before and after the intervention period. For executive function, namely inhibition (the Stroop task) and updating (Letter Updating Task), both cycling groups improved in accuracy after the intervention compared to non-cycling control participants. E-bike participants also improved in processing speed (reaction times in go trials of the Stop-It task) after the intervention compared to non-cycling control participants. Finally, e-bike participants improved in their mental health score after the intervention compared to non-cycling controls as measured by the SF-36. This suggests that there may be an impact of exercising in the environment on executive function and mental health. Importantly, we showed a similar (sometimes larger) effect for the e-bike group compared to the pedal cyclists. This suggests that it is not just the physical activity component of cycling that is having an influence. Both pedal cycles and e-bikes can enable increased physical activity and engagement with the outdoor environment with e-bikes potentially providing greater benefits.

## Introduction

Healthy ageing has been defined as the process of developing and maintaining functional ability, the ability to perform tasks of daily living, that enables well-being in older age [1]. Successful ageing [2] is the conception that ageing is not necessarily accompanied by decline in cognitive function and reduction in brain matter. Although a number of these processes show the greatest rate of decline in late adulthood, they have been demonstrated to be highly malleable, even in older age [3].

Recent intervention studies have demonstrated that cognitive function and brain integrity can be maintained, or even improved, through increasing the frequency and duration of moderate to vigorous exercise (e.g., [3, 4, 5]) even over a short period of time (e.g., six weeks in a younger population; [6]). Exercise can also reduce the occurrence of age-associated neurodegenerative disorders, such as Alzheimer’s disease and vascular dementia [7, 8, 9]. Studies focusing on the effect of exercise in late adulthood have shown improvements in cognitive function. Colcombe and Kramer (2003) [10] reported that exercise broadly improves cognitive function in older adults across a number of domains with medium to large effect sizes. These include executive function (tasks such as coordination, inhibition, planning, and working memory [11]), controlled abilities (tasks that require controlled, effortful processing which can become automatic with consistent practice), speed (e.g., simple reaction time or finger tapping) and spatial functioning (e.g., spatial reasoning), with the strongest effect size observed for executive function. Executive function-related processes and the brain areas that support them have been shown to be disproportionately sensitive to ageing [12]. These, however, appear to be the most amenable to exercise interventions relative to other cognitive functions, particularly aerobic exercise [13].

Exercise improving cognitive function is possibly related to increasing cerebral vascular blood flow [14] enabling increase in volume and cell regeneration in regions of the brain that are critical for efficient cognitive functioning (e.g., the hippocampus [5, 6]). Thomas and colleagues [6] found increases in anterior hippocampal volume in sedentary, young to middle-aged (average age 34) adults after a 6-week exercise (30 minutes of cycling on an exercise bike, 5 days a week) intervention [6]. Therefore, exercise, specifically aerobic exercise on a bicycle, may improve cognitive function through vascular brain changes.

In addition to direct effects on cognitive function, exercise can protect cognitive performance against the adverse effects of lower well-being [15] as evidence suggests that exercise increases subjective, or psychological, well-being [16]. It has been found that exercise improves self-efficacy and well-being improvement overall [8]. Furthermore, significant associations have been found between self-rated meaningfulness of life and regular and intensive physical exercise [17]. The large effect of physical activity on view of self and global well-being are explained by change in self-efficacy and belief in ability to achieve requisite actions in order to satisfy situational demands. This was found to be the most salient variable affecting well-being and psychological health. If psychological well-being is increased through exercise it may also aid cognitive function in older adults as cognition has been shown to be associated with variation in mean levels of well-being in older adults and lower levels of depression [15].

Cognitive function and well-being have also been shown to increase from simply being outside in the environment (e.g., [18, 19]). This could be related to the outdoor environment having the potential to promote attention restoration, stress reduction, and the evocation of positive emotions [18]. Exercising outside in the environment, for example cycling, as opposed to indoor exercise (e.g. in a gym setting) could, therefore, further augment the already demonstrated benefits of physical activity for cognitive function and well-being. However, very few studies have investigated the impact of exercising in the outdoor environment. For those studies that have been conducted, it has been found that green exercise can led to a significant improvement in self-esteem, positive mood and general increase in well-being [18, 19, 20]. In addition to effects of aerobic exercise on cognitive function, particularly executive function, outdoor exercise such as cycling, requires navigation in the environment, enabling changes in brain regions supporting spatial encoding [5, 6, 21].

Given the associations between cognitive function, well-being and exercise in older adults, the aim of the study reported in this paper was to investigate the effect of cycling outdoors on cognitive function and mental health and well-being of older adults (over 50 years old). Compared to other studies, which measure effect of indoor exercise (e.g., [3, 5, 6, 13, 14]), we wanted to investigate cycling as a form of aerobic exercise in its natural environment, i.e. outdoors. We expected aerobic exercise to be an additional benefit to cognition and well-being on top of the effect of being outdoors. In particular, we were interested in whether there was a difference in performance between users of assistive technology in the form of electric bikes (‘e-bikes’) to cycle outdoors in the environment and regular pedal cycle users, with pedal cycling requiring more aerobic exertion than e-bikes.

Based on prior work on cognitive benefits of exercise (as reviewed above), we predicted that in particular executive function (i.e., inhibition, updating, task switching, and working memory processes) would be improved by increased exercise during the eight-week intervention. Such an effect would be demonstrated by improved post intervention scores compared to baseline (pre intervention), with this improvement being greatest in the pedal than e-bike cyclists (due to the additional physical exertion required) and compared to non-cycling controls. We also expected to find an increase in spatial reasoning tasks, and in positive mood and well-being after the interventions.

## Method

### Participants and study design

In this study we recruited non-cyclist older adults (over the age of 50) to take part in a cycling intervention, measuring cognitive performance, mental health and well-being before and after the intervention. This study was part of a wider ‘cycleBOOM’ project (www.cycleboom.org) that aimed to develop a better understanding of how the design of the built environment and technology shaped engagement with, and experience of, cycling as people get older, and how this affected their independent mobility, health and well-being. Older adults were asked to cycle for an eight-week period. This intervention length has been successful in improving cognitive and brain function in prior work (e.g., [6, 10]).

We tested a total of 100 older adults (this was based on a medium effect size, as reported in the studies included in Colcombe and Kramer's, 2003, meta-analysis [10], for mixed ANOVA with interactions: effect size = .04 power = .80, alpha = .05, minimum total sample size = 111), 26 of which were non-cycling controls, 36 were pedal cyclists and 38 were e-bike participants (see Table 1 for demographic information for the three groups). We recruited older adults (over the age of 50) who reported that they had either done no cycling in the past five years, or that their cycling had seriously diminished over that period. Participants first undertook a cycle training assessment/skills development program with an accredited trainer. The timing between the training and the baseline assessment was typically a week and in no case no longer than a month before the trial started. The non-cycling controls were included to measure practice effects, and to assess whether the intervention effect was greater than this, and did not undertake the cycle training. Participants were pseudo-randomly assigned to one of three groups: pedal cycling, e-bike or non-cycling control groups. Priority was given to filling the cycling spots then controls were recruited to match sample characteristics (age and sex; see demographic information in Table 1) as well as trial season. The control group were recruited after the experimental groups had started to be run so that we could match age and gender in the control group with those participants in the experimental groups. The control group were aware that they would not be cycling during the trial and those in the experimental group were all re-engaging with cycling. Cycling was completed in the Reading and Oxford areas. Informed written consent was obtained from all subjects in accordance with the Declaration of Helsinki and ethical approval was obtained from the University of Reading's Research Ethics Committee (Registration No: 14/31) and the research complied with the ethical requirements of Oxford Brookes University (Registration No: 140813).

**Table 1:** Demographic information (mean age, gender, mean years in education [YiE], mean pre-intervention mini-mental state examination [MMSE] score, mean pre-intervention physical activity scale for the elderly [PASE]) for the non-cycling controls, pedal cyclists and e-bike cyclists.

Group	Age (*SD*, *range*)	Gender
Non-cycling Controls	66.04 (8.84, 50–82)	19 Females
Pedal Cyclists	63.03 (7.47, 50–83)	20 Females
E-bike Cyclists	61.90 (7.00, 50–82)	20 Females

Note: The three groups did not differ in age or gender composition, *F* (2, 97) = 2.31, *p* = .105; *F* (2, 97) = 1.89, *p* = .156, respectively.

Prior to commencing the trail participants completed a battery of cognitive tasks and well-being questionnaires (see descriptions below) as well as providing demographic information including their age, gender, and how many years they had spent in education. Demographic information was obtained prior to the trial starting and the experimenter was unblinded to group allocation. The cognitive testing battery employed included tasks assessing executive function, spatial reasoning and memory. The instructions for the tasks were standardised, read from a script and presented on the screen for the participant to also read for all the computerised tasks. The pedal and e-bike cyclists were asked to cycle at least three times a week for thirty minutes in duration for each cycle ride for an eight-week period. Pedal cyclists could either use their own cycle for the trial or they were loaned one by the study. All e-bike participants were provided with the same Raleigh (Motus) e-bike to complete the trial. E-bike participants cycled in the outdoor environment like their pedal cycling counterparts but had electrical assistance from a motor on the pedal bike (a choice of five settings; 'off', 'eco', 'tour', 'sport', 'turbo', each increasing the amount of electrical assistance the participant gained from the motor on the e-bike). All participants were asked to maintain their normal level of physical activity during this period and control participants were asked not to participate in any outdoor or indoor cycling activity. All participants completed daily diaries during the intervention documenting the type and duration of exercise they conducted. All participants came back to complete the cognitive tests and well-being questionnaires again after they completed the intervention, eight weeks later. The assessment was done no sooner than one week before the start of the trial and no longer than one week after the end of the trial. An online exit survey (via google docs) containing 12 questions about their cycling behavior since completing the trial was also conducted several months after their trial had finished, gauging the extent to which participants were still engaged in cycling. This survey was completed by 73 participants.

### Cognitive and well-being battery

The following tests were administered before and after the eight-week period. These tasks were presented in a counterbalanced order across participants.

### Executive function tasks

#### Verbal fluency

This task measures executive function, specifically updating [22]. The verbal fluency task was split into two parts, the letter verbal fluency (where participants are required to say as many words beginning with the letter ‘F’ in one minute) and the category verbal fluency (where participants are required to say as many different animals in one minute). Responses were recorded and written down by the experimenter. The score reflects the number of words that the participant stated within the allocated time.

#### Plus-minus task

This measures task switching [23]. The plus-minus task is a pen-and-paper task. Participants had to add three to a series of numbers (30 numbers on each page) on the first page, subtract three from each number on the second page and then alternate between adding three and subtracting three on the last page. Time taken to complete each section and accuracy was recorded for this. The interference from completing the switching task was calculated by deducting the average of the first two sections.

#### Letter updating task

This task measures working memory and updating [23, 24]. The participant was required to remember a sequence of letters. Letters were presented serially on the screen for 2000 ms with a 150 ms blank screen in between letter presentation. The sequence ranged from four to nine letters in length. The participant was required, for each letter that appeared on the screen, to state the previous two letters (that are no longer present on the screen) and the one that was currently on the screen in the order that they appeared. When the sequence was complete, the participant heard a beep and had to repeat, again, the last three letters they saw. The participant had two practice trials before the experimental trials commenced. The total score correct was the number of trials the participant got the last three letters correct of the twelve trials presented.

#### Stroop task

This task measures inhibition [23, 25]. Words are presented on the screen one at a time. The words are colour words ‘green’, ‘blue’, ‘red’, ‘yellow’ that are printed in these different coloured inks. The participant is required to press a key with the colour indicated on for the ink colour, inhibiting the written word presented. Each word was displayed for 1300 ms with a 350 ms blank screen in between word presentation. There were 36 practice trials (22 congruent trials {written word and ink colour the same}, 14 incongruent trials {written word and ink colour different}) and 72 experimental trials (36 congruent, 36 incongruent). The interference from reporting the ink colour and not the written word was calculated by subtracting the average incongruent accuracy from that of the congruent trials.

#### Stop-it task

This task measures inhibition and was designed by Verbruggen, Logan, and Stevens (2008) [26]. It is a version of a stop signal task. A square or a circle is displayed on the screen for 1250 ms. Participants were instructed to respond as quickly as possible to the identity of the stimulus (1.6 cm × 1.6 cm) by pressing the \ (square) or / (circle) keys, which depicted that object on them as a memory aid. On 25% of trials (stop trials), participants heard a tone through the speaker of the laptop that indicated that they should withhold their response on that trial. The tone was initially presented 250 ms after the visual stimulus appeared, and was adjusted using a tracking procedure by which the latency increased by 50 ms following a successfully withheld response, and decreased by 50 ms following a failure to withhold a response. Participants completed 600 trials in total (75% go). The primary measures are Stop-Signal Reaction Time (SSRT) and go RT.

#### Eriksen flanker task

This task measures inhibition and is an arrow version of the original Eriksen flanker task [27]. During this task participants are presented with a series of five arrows presented in a row on the screen at the same time, each pointing left or right. A set of five arrows was displayed for a duration ranging between 1100 and 1500 ms with no blank screen in between sets of arrows. A central fixation cross was displayed for 2000 ms before the first set of arrows appeared. Participants had to respond as quickly as possible the direction of the middle arrow (right or left, using corresponding arrows on the keyboard), ignoring the external arrows. There were 100 trials in total, with no practice session. On 50 trials, all the arrows pointed in the same direction as the middle arrow (congruent trials). On 50 of trials, the external arrows pointed in a different direction to the middle arrow (incongruent). The interference from reporting the direction of the middle arrow and not the external arrows was calculated by subtracting the average incongruent accuracy from that of the congruent trials.

### Memory tasks

#### Consortium to Establish a Registry for Alzheimer's Disease (CERAD) immediate and delayed recall

The CERAD [28] task measures immediate and delayed memory recall. The immediate task required the participant to recall as many of the ten words they have just heard. There are three trials of the same words in different orders. The delayed test was completed after the plus-minus task, where participants had free-recall and a recognition test (10 of 20 words presented were in the original list). The immediate recall was scored out of 30 (3 lists of 10 words), as was the delayed recall (10 for free recall and 20 for the recognition test).

#### MMSE

The MMSE [29] measures memory and orientation, with questions such as "what is today's date?" and was scored out of 30.

### Spatial function tasks

#### Mazes and mental rotation task

These tasks were designed in house by the authors and the mental rotation tasks were similar to stimuli employed to assess this ability in other studies [30,31,32]. These stimuli are included in S1 Fig. Five mazes were completed, varying in difficulty. The participant had to find the route from the 'start' indicated on the maze to the 'end' as quickly as possible. They were timed during completion and number of errors (deviation from the correct route) were scored. The mental rotation task contained five 3-D mental rotation trials, whereby the participant had to match the shape from three options (two incorrect), which only differed in orientation, to the original. There were also five 2-D trials.

### Well-being, physical activity and health questionnaires

#### Psychological Well-Being (PWB) questionnaire

Developed by Ryff (1995) [33] this questionnaire measured 6 dimensions of well-being: autonomy, environmental mastery, personal growth, positive relations with others, purpose in life, and self-acceptance. The 84-items are presented on a 6-point Likert scale (1 = strongly disagree, 6 = strongly agree), with reverse coding on some items and a high score reflecting higher well-being (Cronbach’s α pre/post = 0.95/0.95).

#### Satisfaction in Life (SL)

Measuring life satisfaction, participants stated how much they agreed with five statements on beliefs of the conditions of their life [34]. A 7-point Likert scale, with 1 = strongly disagree and 7 = strongly agree (Cronbach’s α pre/post = 0.90/0.86).

#### Positive and Negative Affect Scale (PANAS)—general

This measure, developed by Watson, Clark and Tellegen (1988) [35], included 20 adjectives which participants had to rate on the extent they felt these adjectives generally (e.g., over the last week) on a five-point Likert scale (1 = very slightly or not at all, 5 = extremely). There are 10 positive adjectives (e.g., proud, interested, excited) and 10 negative adjectives (e.g., distressed, upset, guilty). The positive and negative scores are summed separately (Cronbach’s α positive PANAS pre/post = 0.85/0.81, negative PANAS pre/post = 0.89/0.88).

#### Health survey short form (SF-36)

This is a short questionnaire designed to measure physical and mental health [36]. A high score reflects greater health and there are mental and physical health sub-scales (Cronbach’s α mental health pre/post = 0.90/0.86, physical health pre/post = 0.92/0.92).

#### Physical Activity Scale for the Elderly (PASE)

This is a brief survey designed specifically to assess physical activity in epidemiological studies of older adults [37]. The PASE score combined information on leisure, household and occupational activity.

### Daily diary

The daily diary was designed by the team to capture information about the participants' cycle rides they completed and any other physical activity they took part in during the eight-week intervention (e.g., walking, sports, other physical activities). Each day, participants were asked to fill in what time they started and completed any physical activity, the purpose of the activity (if cycling or walking somewhere) and where they went from and to. For a measure of cycling time to be calculated, participants recorded the start and end time of each cycle and this time spent cycling was summed across the week and then averaged over the eight-week period to provide an average weekly cycling duration.

### Data analysis

IBM SPSS Statistics 25 package was used to analyse the data. Any missing data and outliers (1.5*IQR for that group) for each task were removed and replaced with the mean of the group. Missing data points and outliers were minimal within the dataset, which were subsequently imputed with the means of the group, namely < .007% of the total number of observations that were missing and < .01% of the total number of observations that were outliers. The number of outliers, *χ*^*2*^ (2) = 3.00, *p* = .223, and missing data frequencies, *χ*^*2*^ (2) = 6.00, *p* = .199, did not differ between the three participant groups. A composite score for executive function was intended to be used in the analyses. However, the relevant measures at baseline did not significantly correlate (see S1 Table) and thus it would be meaningless to calculate composite scores. The mental rotation time and average time taken to complete the mazes significantly correlated and so were also combined to form a single Spatial Function Time composite score (see S2 Table). As mental rotation accuracy and average maze errors did not significantly correlate, separate analyses were conducted on these measures, Bonferroni corrected (alpha level, *p* < .017). For the memory tasks, the CERAD immediate and delayed recall significantly correlated (see S3 Table) and so were combined to form a composite CERAD score which was used in subsequent analyses.

For all measures (executive function, memory, spatial function, questionnaires), 2 (session; before intervention, after intervention) x 3 (group; non-cycling controls, e-bike cyclists, pedal cyclists) mixed method Analysis of Variance (ANOVAs) were conducted to investigate the impact of cycling on cognitive function.

## Results and discussion

### Group composition

The three groups did not differ in years in education, MMSE or pre-intervention physical activity level, *F* (2, 97) = .77, *p* = .466, *F* (2, 97) = 1.93, *p* = .151, *F* (2, 97) = .98, *p* = .378, respectively (see Table 2). PASE did not differ significantly between groups after the intervention either, *t*(98) = 1.547, *p* = .125 (control *M* = 35.62, *SD* = 21.93, cyclist *M* = 44.47, *SD* = 26.09). 72% of participants completed the trial during the warmer months to maximise adherence to the trial. Furthermore, E-bike cyclists (*M* = 1.86, *SD* = 1.76), pedal cyclists (*M* = 2.22, *SD* = 1.76) and non-cycling control participants (*M* = 1.92, *SD* = 1.65) did not differ in the frequency of doing these other activities, *H* (2, 97) = 1.08, *p* = .582, as reported in the PASE. E-bike cyclists (*M* = 1.53, *SD* = 1.41), pedal cyclists (*M* = 1.58, *SD* = 1.25) and non-cycling control participants (*M* = 1.62, *SD* = 1.65) also did not differ in the number of other activities they participated in, *F* (2, 97) = .033, *p* = .968. Finally, E-bike cyclists (*M* = 2.50, *SD* = 2.47), pedal cyclists (*M* = 2.58, *SD* = 2.39) and control participants (*M* = 2.46, *SD* = 2.32) did not differ in the time spent completing these activities, *H* (2, 97) = .049, *p* = .976, as reported in the PASE. Participants continued to report their other physical activities (additional to cycling) that they conducted throughout the cycling trial (complete diaries received, *N* = 81). Again, the average time spent completing other activities did not differ (E-bike [*M* = 1.21, *SD* = .54], pedal [*M* = 1.21, *SD* = .38] and control participants [*M* = 1.27, *SD* = .30]) across the participant groups, *F* (2, 78) = .141, *p* = .869).

**Table 2:** Years in education (YiE), mini-mental state examination (MMSE) and physical activity for the elderly results for the non-cycling controls, pedal cyclists and e-bike cyclists before the trial commenced.

Group	YiE (*SD*)	MMSE	PASE (*SD*)
Non-cycling Controls	15.94 (1.97)	27.58 (1.21)	35.23 (17.25)
Pedal Cyclists	16.83 (3.89)	26.86 (1.90)	40.86 (24.84)
E-bike Cyclists	15.98 (3.57)	26.97 (1.20)	43.72 (21.87)

### Cycling statistics during the trial

Participants kept a diary of their cycling activity during the trial and recorded the duration of each journey. We found e-bike cyclists spent marginally more time cycling on average each week than pedal cyclists, *t*(72) = 1.80, *p* = .076 (see Table 3 for Means and SDs). This is likely due to the ease associated with cycling with a motor, enabling the e-bike participants to cycle for longer periods of time. This indicates that e-bikes, due to supporting the cycling, may enable increased activity and durations of cycle rides. Many of the participants commented that they felt they could go further on the e-bike as they could rely on it to get home if they could not manage it by themselves (see [38] for a qualitative account of factors affecting cycling behavior in ‘Older people’s microadventures outdoors on (e-) bikes’).

**Table 3:** Average weekly (standard deviation) cycling durations for the pedal and e-bike cyclists.

Group	Average Hours spent Cycling Each Week (*SD*)	Average Number of Weeks Cycled for
Pedal Cyclists	2.07 (0.59)	8.34 (0.71)
E-bike Cyclists	2.39 (0.90)	7.93 (1.36)

Note: Whilst some cyclists extended their cycling into the 9th week just prior testing, average number of weeks the participant groups cycled for during the intervention did not significantly differ, *t*(72) = -1.59, *p* = .117.

Additionally, e-bikers spent on average 26% of the time in the highest motor setting (turbo; *SD* = 34), 7% in the next highest setting (sport, *SD* = 11), 24% in tour (*SD* = 22), 28% in eco (*SD* = 26), and 15% (*SD* = 26) with the motor off. This means that on average for only 15% of their cycling time, e-bike participants were not using the motor to aid their cycling, thus being comparable to pedal cyclists.

Participants (pseudonym) often included comments in their daily diary of cycling experience summing up their belief of the contribution to psychological well-being, for example:

*"On Sunday I took the (E-)bike out for the afternoon to cheer myself up*. *Gloomy day but the countryside around is lovely so felt better when I came back*!*"* (Alysia)

*“After a stressful morning I had time to unwind on the (E-)bike*.*”* (Christopher)

### Executive function measures

Large effect sizes for improvement in executive function after exercise have been demonstrated [10] and we predicted an increase in executive function in the cycling groups, thus would expect significant interactions between group and session, with the control group not improving after the intervention. The ANOVA demonstrated that there was no interaction for verbal fluency (*F*(2, 97) *=* .739, *p =* .480), plus-minus task (*F*(2, 97) *=* 4.07, *p =* .667), letter updating (*F*(2, 97) *=* 1.92, *p =* .152), Eriksen interference score (*F*(2, 97) *=* .623, *p =* .538), all measuring executive function.

**Fig 1:**
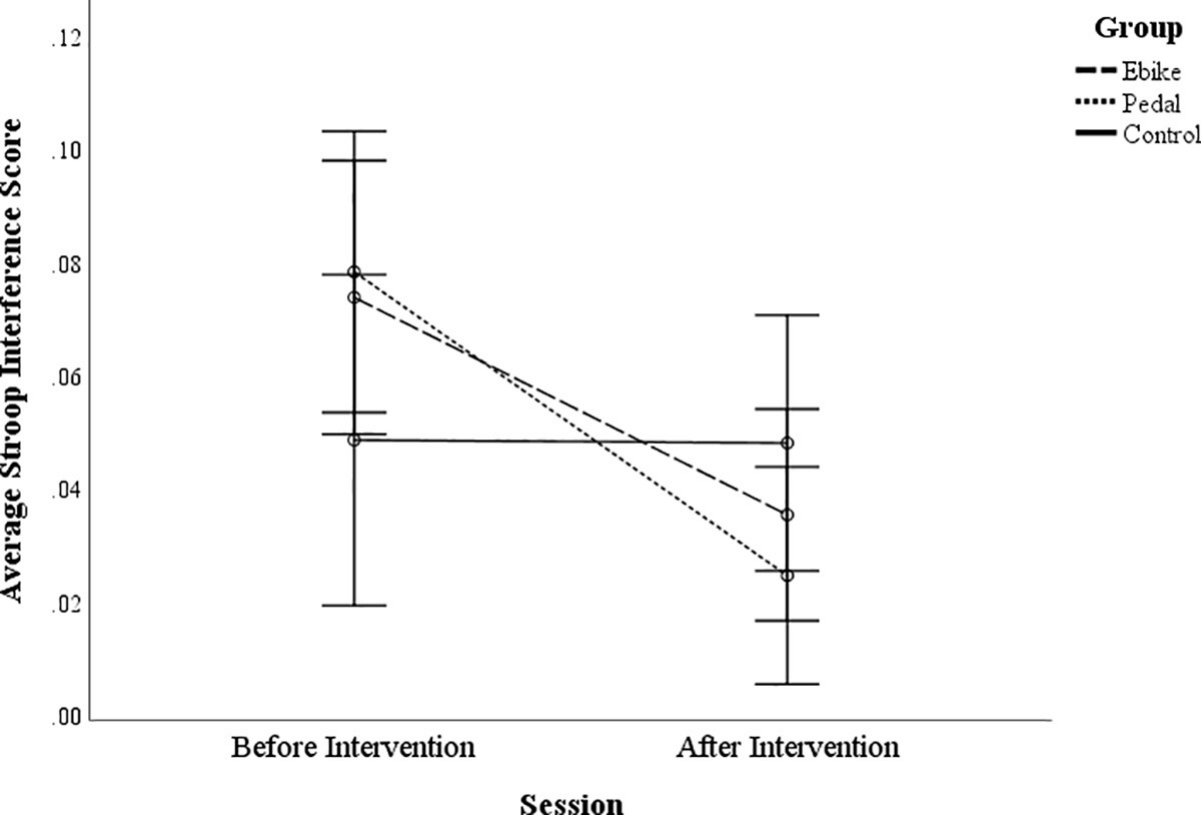
Stroop interference score for the non-cycling controls, E-bike cyclists and pedal cyclists before and after the intervention. Error bars are +/- 2 SEs.

There was, however, a significant Group x Session interaction for the Stroop Interference score, a measure of inhibition, *F*(2, 97) = 3.77, *p* = .026, *ƞ*^2^ = .072. The smaller the score, the less interference that the participant experienced from the written word being incongruent with the ink colour that they were reporting. This measure demonstrated improvement in both the cycling groups after the intervention, for e-bike cyclists, *t*(37) = 2.75, *p* = .009 , and for pedal cyclists, *t*(35) = 5.30, *p* = .000, with less interference after the intervention (see Fig 1), which was not the case for non-cycling control participants, *t*(25) = .03, *p* = .974.

**Fig 2:**
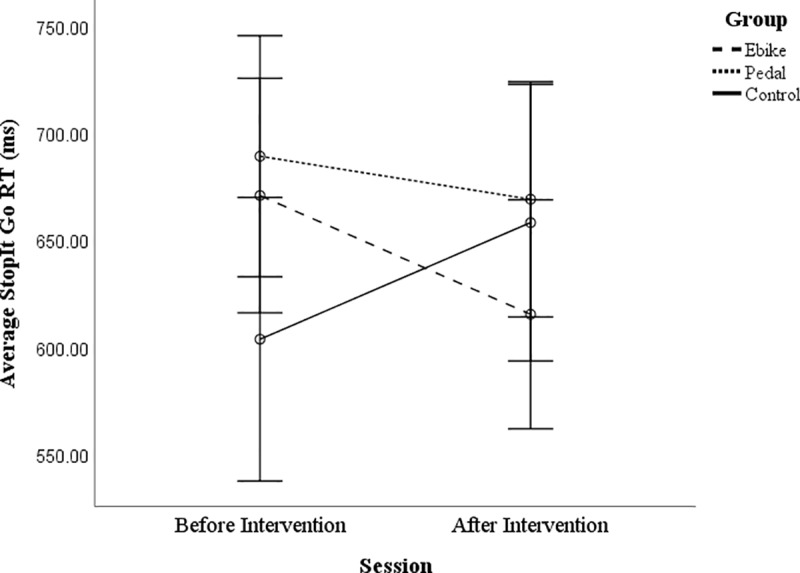
Stop-It task RTs (ms) for go trials for the non-cycling controls, E-bike cyclists and pedal cyclists before and after the intervention. Error bars are +/- 2 SEs.

There was also a significant interaction between session and group for Go RTs in the Stop-It task, a measure of processing speed, *F*(2, 97) = 3.78, *p* = .026, *ƞ*^2^ = .072 (see Fig 2). E-bike cyclists had marginally faster RTs after the intervention compared to baseline, *t*(37) = 1.97, *p* = .056, whereas pedal cyclists did not, *t*(35) = .87, *p* = .391, and the non-cycling controls had a trend towards slower RTs, *t*(25) = -1.75, *p* = .092.

**Fig 3:**
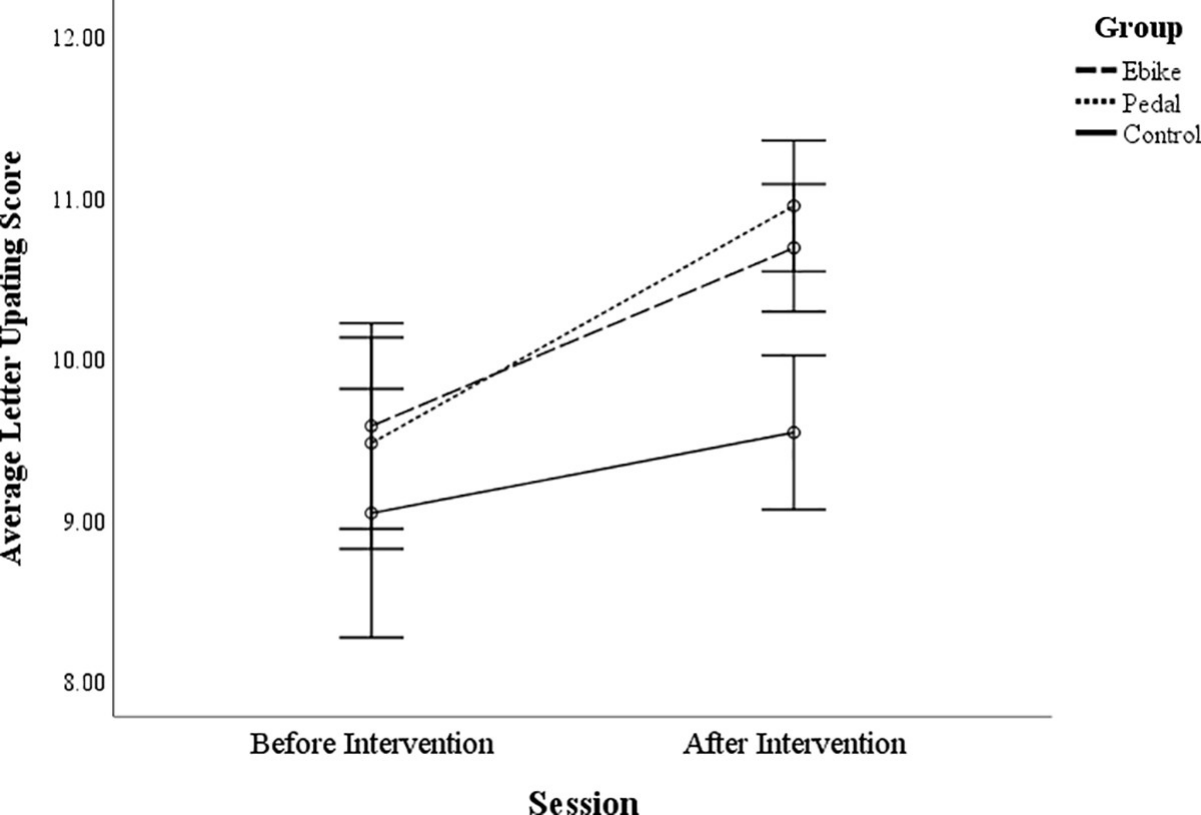
Letter updating score for non-cycling controls, pedal cyclists and E-bike cyclists before and after the intervention. Error bars are +/- 2 SEs.

There was no overall difference between the groups for verbal fluency scores (*F*(2, 97) *=* 2.18, *p =* .119), the plus-minus task (*F*(2, 97) = .368, *p =* .693), Stroop interference scores (*F*(2, 97) = 1.00, *p =* .905), the Eriksen flanker task (*F*(2, 97) *=* 2.28, *p =* .107) or Stop-IT go-RTs, measuring processing speed (*F*(2, 97) *=* .930, *p =* .398). There was a main effect of group for the letter updating task, *F*(2, 97) = 4.20, *p* = .018, with both cycling groups being higher overall in accuracy than the non-cycling control group, *t*(62) = 2.44, *p* = .017 (e-bike cyclists compared to non-cycling controls), *t*(60) = 2.54, *p* = .014 (pedal cyclists compared to non-cycling controls). As there were no significant differences in baseline performance across groups (see S1 File), this main effect is mainly driven by the after intervention accuracy being higher for the cycling groups, *t*(98) = 3.72, *p* = .001 (see Fig 3) as demonstrated by the interaction.

There was no session effect for the plus-minus task (*F*(2, 97) *=* 2.81, *p =* .097) or for the go RTs in the Stop-IT task, *F*(2, 97) *=* .193, *p =* .662. There was a session effect for the groups overall, with improvement after the 8-week period for verbal fluency (*F*(2, 97) = 8.50, *p* = .004, *ƞ*^2^ = .081), letter updating, *F*(2, 97) = 27.41, *p* = .000, *ƞ*^2^ = .221 (see Table 4 for all Means and SDs for the executive function measures), the Stroop interference score (mainly driven by the improvement in the cycling groups after the intervention, as well as practice effects improving performance after the intervention), *F*(2, 97) = 15.96, *p* = .000, *ƞ* ^2^ = .141, and a marginal session effect of the reduction of interference in the Eriksen flanker task, *F*(2, 97) = 3.73, *p* = .056, *ƞ*^2^ = .037. These session effects alone are likely due to performance improving overall after the intervention as a result of practice.

**Table 4:** Group (non-cycling controls, e-bike cyclists, pedal cyclists) means (SDs) for verbal fluency, plus-minus interference score (IS), letter updating, stroop IS, eriksen IS, stop-it, CERAD, mental rotation, maze completion before and after the intervention.

Measure	Non-Cycling Controls		E-bike Cyclists		Pedal Cyclists		Effects
	Before	After	Before	After	Before	After	
**Verbal Fluency**	33.69 (5.64)	36.50 (7.81)	36.63 (8.32)	39.21 (9.21)	38.41 (9.27)	39.31 (6.27)	Significant **Session** effect^1^
**Plus Minus IS**	-1.46 (2.30)	-0.90 (1.44)	-1.24 (1.62)	-.66 (1.23)	-1.18 (2.03)	-1.07 (1.48)	No significant effect
**Letter Updating**	9.04 (2.18)	9.54 (1.63)	9.58 (1.94)	10.68 (.93)	9.47 (1.87)	10.94 (1.17)	Significant **Session** effect^1^ Significant **Group** effect^2^
**Stroop IS**	.05 (.07)	.05 (.07)	.07 (.08)	.04 (.06)	.08 (.07)	.02 (.04)	Significant **Session** effect^1^ Significant **Group by Session** interaction^3^
**Eriksen IS**	.08 (.04)	.06 (.05)	.07 (.06)	.07 (.05)	.06 (.04)	.05 (.04)	No significant effect
**Stop-IT Go RTs**	603.40 (162.32)	657.87 (166.25)	670.50 (180.73)	615.03 (159.69)	688.84 (163.88)	668.77 (172.24)	Significant **Group by Session** interaction^4^
**CERAD composite**	47.35 (4.41)	50.35 (4.88)	46.37 (5.07)	48.74 (5.52)	47.58 (4.46)	49.28 (3.63)	Significant **Session** effect^1^
**Mental Rotation Accuracy**	8.35 (1.09)	8.19 (1.53)	7.37 (1.85)	7.74 (1.70)	7.39 (2.10)	7.42 (1.75)	No significant effect
**Mental Rotation Time (seconds)**	185.34 (72.27)	160.64 (67.23)	191.09 (91.39)	154.95 (51.62)	183.94 (78.08)	181.36 (73.31)	Significant **Session** effect^1^
**Average Maze Errors**	1.95 (.96)	2.22 (1.24)	1.72 (1.05)	1.64 (1.08)	1.96 (.88)	1.71 (1.11)	No significant effect
**Maze Time (seconds)**	118.55 (40.80)	99.94 (16.65)	122.95 (50.03)	100.89 (38.39)	123.13 (38.19)	116.44 (44.89)	Significant **Session** effect^1^

IS = Interference Score

^1^Scores after the intervention indicated better performance

^2^Both cycling groups having better performance overall.

^3^Both cycling groups had better performance than the controls after the intervention

^4^Only the e-bike participants had better performance after the intervention compared to controls.

*NB*. The non-cycling control group, e-bike and pedal cyclists did not significantly differ on any of the test measures at baseline (see S1 File).

### Memory measures

There was no group, session or interaction effect on the MMSE scores, group effect *F*(2, 97) *=* 1.35, *p =* .264, session effect *F*(2, 97) *=* .328, *p =* .568, interaction *F*(2, 97) *=* .616, *p =* .542. The CERAD composite did not show an effect of cycling on recall either, group effect *F*(2, 97) *=* .874, *p =* .420, interaction *F*(2, 97) *=* .495, *p =* .611, which is not surprising given the lower effect sizes reported for exercise interventions on memory. There was a session effect for the CERAD composite score, *F*(2, 97) = 30.84, *p* = .000, reflecting better performance after the intervention, *t*(99) = -5.54, *p* = .000, *ƞ*^2^ = .241, likely due to practice effects.

### Spatial function measures

Despite evidence to suggest there are medium effect sizes for spatial function improvement after exercise [12] and we predicted an increase in this ability in the cycling groups, the ANOVA demonstrated that there was no interaction or group effect for the Mental Rotation Task Accuracy, group effect *F*(2, 97) *=* .874, *p =* .420, interaction *F*(2, 97) *=* .874, *p =* .420, Maze Errors, group effect *F*(2, 97) *=* .874, *p =* .420, interaction *F*(2, 97) *=* .874, *p =* .420, and Spatial Function Time (Composite of the completion times for the mazes and mental rotation task), group effect *F*(2, 97) *=* .874, *p =* .420, interaction *F*(2, 97) *=* .874, *p =* .420. Again, there was a significant session effect in the Spatial Function Time composite indicating the influence of practice on increased speed from completing the tests again after the intervention, *F*(2, 97) = 10.62, *p* = .002, *ƞ*^2^ = .099.

### Well-being and mental health questionnaires

As with spatial function and some of the executive function measures, we predicted to see an increase in well-being in the cycling groups compared to controls. The ANOVA demonstrated that there was no interaction, or group effect for the PWB, group effect *F*(2, 97) *=* .441, *p =* .644, interaction *F*(2, 97) *=* 1.48, *p =* .232, session effect, *F*(2, 97) *=* 1.95, *p =* .166, the SL, group effect *F*(2, 97) *=* 1.03, *p =* .363, interaction *F*(2, 97) *=* .340, *p =* .713, PANAS positive, group effect *F*(2, 97) *=* 1.95, *p =* .148, interaction *F*(2, 97) *=* 1.01, *p =* .370 or PANAS negative, group effect *F*(2, 97) *=* 1.52, *p =* .223, interaction *F*(2, 97) *=* 1.22, *p =* .300, and session *F*(2, 97) *=* 1.09, *p =* .742, (see Table 5 for *M*s and *SD*s for all well-being measures). There was a session effect for Positive PANAS items, demonstrating an increase in the positive score in all groups after the intervention period, *F* (2, 97) = 8.92, *p* = .004, *ƞ*^2^ = .072. This was also the case for the SL, *F*(2, 97) = 8.32, *p* = .005, *ƞ*^2^ = .079.

**Table 5:** Group (non-cycling controls, e-bike cyclists, pedal cyclists) means (SDs) for psychological wellbeing (PWB), positive and negative affect scale positive score (PANAS-P) and negative score (PANAS-N), SF-36 mental (SF-36 mental) and physical health (SF-36 physical) and life satisfaction (SL).

Measure	Non-Cycling Controls		E-bike Cyclists		Pedal Cyclists		Effects
	Before	After	Before	After	Before	After	
**PWB**	4.83(.56)	4.79(.57)	4.72(.51)	4.81 (.50)	4.81 (.46)	4.92 (.45)	No significant effects
**PANAS-P**	33.80 (5.94)	35.12 (4.53)	33.97 (4.94)	35.90 (4.40)	36.19 (4.61)	36.75 (4.25)	Significant **Session** effect^1^
**PANAS-N**	14.35 (4.80)	14.35 (4.75)	14.92 (5.94)	13.91 (4.18)	12.69 (2.39)	13.26 (4.01)	Significant **Session** effect^1^
**SF-36 Mental**	79.34 (13.45)	77.91 (11.63)	76.90 (13.94)	81.81 (8.20)	80.61 (11.89)	83.01 (10.19)	Significant **Session** effect^1^ Marginal **Group by Session** interaction^2^
**SF-36 Physical**	73.02 (17.17)	74.70 (11.63)	77.51 (14.52)	81.49 (13.93)	80.49 (12.16)	81.94 (11.24)	Significant **Session** effect^1^
**SL**	25.39 (6.17)	26.00 (5.63)	26.50 (4.78)	27.76 (3.39)	26.81 (4.73)	27.69 (4.74)	Significant **Session** effect^1^

^1^ Better performance after the intervention for all groups

^2^ E-bike participants had better performance after the intervention compared to controls.

**Fig 4:**
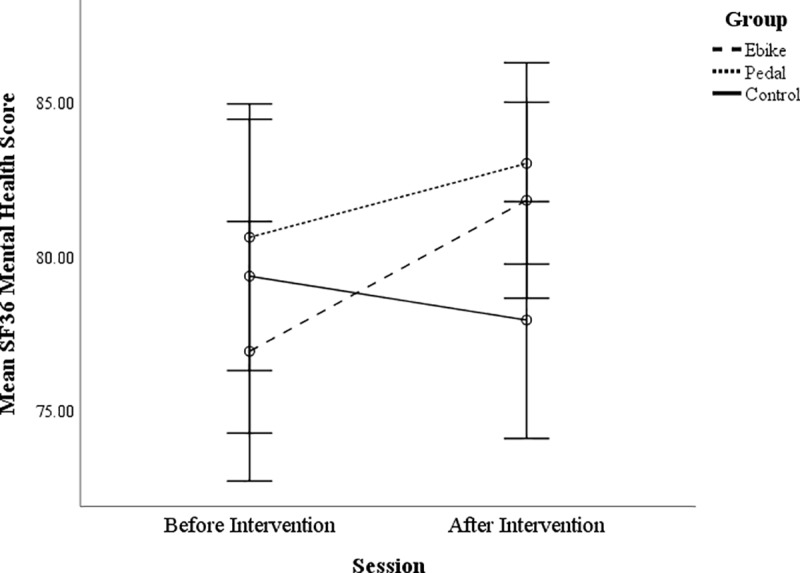
SF-36 mental health component for the non-cycling controls, e-bike cyclists and pedal cyclists before and after the intervention. Error bars are +/- 2 SEs.

There was also a marginal interaction between session and group for the mental health component of the SF-36, *F*(2, 97) = 4.25, *p* = .017, *ƞ*^2^ = .081, with the e-bike cyclists increasing in this score, *t*(37) = -3.45, *p* = .001, but pedal cyclists and non-cycling controls not, *t*(35) = 1.56, *p* = .128, *t* (25) = 1.03, *p* = .311 (see Fig 4). There was also a significant session effect (with an increase in their mental health score after the intervention period), *F*(2, 97) = 5.13, *p* = .026, *ƞ*^2^ = .050, but no group effect, *p* > .05, *F =* .78. This interaction was not the case for the physical health component of this measure but there was a session effect, *F*(2, 97) = 7.74, *p* = .007, *ƞ*^2^ = .072.

We checked the extent to which more time spent cycling was associated with stronger improvement in cognitive performance. There was no cycling dose effect on any of the measures that showed improvement from cycling, so more cycling overall did not relate to greater improvement in cognitive function or well-being (see S3 File).

## Discussion

The aim of this study was to investigate the effect of cycling in the outdoor environment on cognitive function, specifically executive function, and well-being in older adults. Participants were asked to cycle for at least an hour and a half each week for an eight-week period, either on a conventional pedal bike or an electrically assisted e-bike. We expected to find increased accuracy in a number of different executive function tasks and well-being in the cycling groups after the intervention compared to baseline and the non-cycling control group.

In line with our predictions, we found trends for improvement in executive function in the Stroop task and letter updating task in both cycling groups compared to baseline and the non-cycling controls. We also found improvement in speed of processing for go trials in the Stop-It signal task only for e-bike participants during the intervention. Measures of memory and spatial functioning did not show an effect of cycling. Furthermore, we found increases in self-reported mental health on the SF-36 health survey for only the e-bike cycling group. Despite strong evidence from previous studies for an increase in well-being after exercise and the impacts of the outdoor environment on this aspect of mental health, we did not find increases on the PWB, SL or PANAS questionnaires.

The increase in inhibition and updating suggests that there may be an impact of exercise on executive function. These results support the notion that even cognitive processes that show the greatest rate of decline (e.g., executive function, processing speed) remain malleable [3, 4, 5, 6, 9]. The fact that we found effects in the Stroop but not the Eriksen task suggests that these tasks are tapping into slightly different processes or have different levels of sensitivity, especially to individual differences, with the latter being more plausible [39]. Hedge et al. (2017) [39] found that the reliabilities for the Eriksen flanker task measure ranged from .37 to .74. Change due to a short exercise intervention was demonstrated in the more sensitive tasks of executive function as per Hedge et al. (2017) [39], namely the Stroop and Letter Updating task, in the current study.

Executive function aside, exercise has also been shown to increase psychological well-being [8] and this has the potential to aid maintenance of cognitive function as low well-being and depression have been linked to poor cognitive function (e.g., [15, 40]). We found an increase in the mental health component of the SF-36 for e-bike participants, but not an increase in well-being on the PWB or SL, or positive affect measured by the PANAS for either cycling group. This was surprising as cycling participants highlighted an increase in their mood and satisfaction from cycling regularly during the intervention through their diary entries. It may be that these measures are not sensitive to change over time since they are designed to capture trait elements that are unlikely to vary, especially over the short period of the intervention, or even as a function of the intervention (e.g., [41]). The mental health component of the SF-36 captures individual’s responses to how much any emotional problems have interfered with their daily and social activities, rather than their subjective opinion on their psychological well-being. This survey has demonstrated high sensitivity and convergent validity for mental health issues [42]. Therefore, the SF-36 may be more proficient at detecting change over, even short periods of, time [42] compared to those measures that we employed that identify more stable traits (e.g., PWB).

An online exit survey was also conducted several months after their trial had finished, gauging the extent to which participants were still engaged in cycling; completed by 73 participants. As participants did not all complete the trial at the same time, participants completed this exit interview at different points in relation to trial completion. Over two-thirds thought that their wellbeing had improved a little or a lot compared to before they took part in the trial and that they had become more physically active. Also, 58% reported that they had cycled and intended to increase or maintain their level of cycling, and a further 27% reported that they had stopped but were actively planning to start cycling again.

Importantly, we showed an equal (if not larger) effect for the e-bike group as well as the pedal cyclists on measures of executive function and well-being. This suggests that it is not just the physical activity component of cycling that aids executive function. E-bikes require less physical exertion than the pedal bikes and often are more rewarding for participants to cycle as they can travel longer distances without having to worry about not being able to get back, cover greater distance in less time, enable coping with physical ailments that make ordinary pedal cycling challenging and encourage more cycling time (as demonstrated by our e-bike participants on average spending longer cycling each week on average). In addition, the novelty of being provided with an e-bike may have increased any effect on cognitive function and well-being. Increasing older adults' independence and mobility, reducing isolation and depression, is likely to have a positive impact on their mental health and cognitive function [43].

We also note that there are limitations to the current study. Due to its high ecological validity, participants cycling in the natural environment at their own discretion, some participants cycled above and beyond that which was required, whereas others just met the required cycling time each week thus adding variability to the data. The control group also, as mentioned previously, increased their physical activity somewhat due to this being monitored, which may have reduced the sensitivity of the effects we found of cycling on cognitive function. Furthermore, we did not employ a more objective measure of change in fitness, such as oxygen uptake (VO^2^ max).

In summary, we found that cycling over an eight-week period showed trends for improving a number of different executive functions, particularly updating and inhibition as well as processing speed for e-bike participants. As e-bike participants benefitted as much (if not more) than pedal cyclists, this suggests that it is not just the physical component of the activity but a number of different aspects of cycling that can improve cognition and mental health, e.g., engagement with the outdoor environment, independence and mobility. We did not find changes, however, in psychological well-being, which may be due to little change on these measures over the intervention period but we did see trends in the expected direction, particularly pronounced for the mental health component of the SF-36.

E-bikes certainly have the potential to re-engage older adults with cycling and provide a great opportunity to increase physical activity and engagement with the outdoor environment. Cycling in general appears to improve some aspects of cognitive function and mental health, however, controlled trials with more sensitive executive function and well-being tasks are required to determine the extent of these effects and to quantify the individual contributions of each component (e.g., physical activity, outdoor engagement, independence, mobility) to cognitive and well-being improvements in older adults.

## Supporting information

S1 Table Executive function correlations. Correlations between the Executive Function Measures.
(DOCX)

S2 Table Spatial function correlations. Correlations between the Spatial Function Measures.
(DOCX)

S3 Table Memory measure correlations. Correlations between the Memory Measures.
(DOCX)

S1 File Supporting information 1 file. Baseline group differences.
(DOCX)

S2 File Supporting information. Data File
(XLSX)

S3 File Supporting information 3 file. Linear regression on the change scores.
(DOCX)

S1 Fig Supplementary Fig 1. Stimuli used for the Mental Rotation Task and Maze Completion Task.
(JPG)
