# Supplementary material for: The effect of cycling on cognitive function and well-being in older adults
Source: PLoS One. 2019 Feb 20;14(2):e0211779. doi: 10.1371/journal.pone.0211779 (PMC6388745; doi:10.1371/journal.pone.0211779)
Supplement: S1 Table — Correlations between the Executive Function Measures. (DOCX) [file pone.0211779.s001.docx]

**S1 Table**

**Executive function correlations.**

Table S1. *Correlations between the Executive Function Measures.*

As none of the executive function measures significantly correlated, a part from Stop It Go RT and Stroop Interference Score, *r* (100) = .233, *p* = .019 (and not at the corrected level), an executive function composite score was not used in the analyses and separate ANOVAs were conducted on each measure, Bonferroni corrected for the number of tests conducted.

|  |  | Letter Updating Score | Verbal Fluency Score | Plus Minus Interference Score | Stroop Interference Score | Eriksen Interference Score | Stop It Go RT |
| --- | --- | --- | --- | --- | --- | --- | --- |
| Letter Updating Score | Person Correlation  Significance | 1 | .070  .489 | .066  .516 | -.057  .576 | -.113  .263 | -.144  .152 |
| Verbal Fluency Score | Person Correlation  Significance | .070  .489 | 1 | .043  .671 | .117  .245 | .016  .876 | -.155  .124 |
| Plus Minus Interference Score | Person Correlation  Significance | .066  .516 | .043  .671 | 1 | .012  .909 | -.163  .105 | -.046  .651 |
| Stroop Interference Score | Person Correlation  Significance | -.057  .576 | .117  .245 | .012  .909 | 1 | .123  .224 | .233*  .019 |
| Eriksen Interference Score | Person Correlation  Significance | -.113  .263 | .016  .876 | -.163  .105 | .123  .224 | 1 | .063  .532 |
| Stop It Go RT | Person Correlation  Significance | -.144  .152 | -.155  .124 | -.046  .651 | .233*  .019 | .063  .532 | 1 |

N = 100

* Correlation is significant at the 0.05 level (2-tailed).
