# Supplementary material for: The effect of cycling on cognitive function and well-being in older adults
Source: PLoS One. 2019 Feb 20;14(2):e0211779. doi: 10.1371/journal.pone.0211779 (PMC6388745; doi:10.1371/journal.pone.0211779)
Supplement: S2 Table — Correlations between the Spatial Function Measures. (DOCX) [file pone.0211779.s002.docx]

**S2 Table**

**Spatial function correlations.**

Table S2. *Correlations between the Spatial Function Tasks (Average Maze Errors and Time, Mental Rotation Accuracy and Time).*

|  |  | Mental Rotation Accuracy | Mental Rotation Time | Average Maze Errors | Average Maze Time |
| --- | --- | --- | --- | --- | --- |
| Mental Rotation Accuracy | Person Correlation  Significance | 1 | .243*  .015 | -.133  .189 | -.020  .846 |
| Mental Rotation Time | Person Correlation  Significance | .243*  .015 | 1 | .051  .614 | .235*  .018 |
| Average Maze Errors | Person Correlation  Significance | -.133  .189 | .051  .614 | 1 | .148  .141 |
| Average Maze Time | Person Correlation  Significance | -.020  .846 | .235*  .018 | .148  .141 | 1 |

N = 100

* Correlation is significant at the 0.05 level (2-tailed).

As the Average Maze Time and Mental Rotation Times correlated significantly, *r* (100) = .235, *p* = .018, these were Z-scored and combined to form a Spatial Function Time Composite Score. The Mental Rotation Accuracy and Maze Errors were analysed separately as they did not correlate significantly, *r* (100) = -.133, *p* = .189.
