# Supplementary material for: The effect of cycling on cognitive function and well-being in older adults
Source: PLoS One. 2019 Feb 20;14(2):e0211779. doi: 10.1371/journal.pone.0211779 (PMC6388745; doi:10.1371/journal.pone.0211779)
Supplement: S3 Table — Correlations between the Memory Measures. (DOCX) [file pone.0211779.s003.docx]

**S3 Table**

**Memory correlations.**

Table S3. *Correlations between Memory Tasks (CERAD Immediate and Delayed Recall and MMSE).*

|  |  | CERAD Immediate | CERAD Delayed | MMSE |
| --- | --- | --- | --- | --- |
| CERAD Immediate | Person Correlation  Significance | 1 | .534**  .000 | .141  .162 |
| CERAD Delayed | Person Correlation  Significance | .534**  .000 | 1 | .125  .214 |
| MMSE | Person Correlation  Significance | .141  .162 | .125  .214 | 1 |

N = 100

** Correlation is significant at the 0.01 level (2-tailed).

As CERAD immediate and delayed recall significantly correlated, *r* (100) = .534, *p* = .000, these were combined into a CERAD composite score which was used in subsequent analyses. MMSE was analysed separately.
