# Supplementary material for: The effect of cycling on cognitive function and well-being in older adults
Source: PLoS One. 2019 Feb 20;14(2):e0211779. doi: 10.1371/journal.pone.0211779 (PMC6388745; doi:10.1371/journal.pone.0211779)
Supplement: S1 File — Baseline group differences. (DOCX) [file pone.0211779.s004.docx]

**Supporting Information 1 File**

**Baseline group differences.**

There were no significant differences on any of the measures between the three groups at baseline; Letter updating task, *F* (2, 97) = 1.01, *p* = .368; CERAD Composite, *F* (2, 97) = .73, *p* = .484; MMSE, *F* (2, 97) = .91, *p* = .407; Verbal Fluency, *F* (2, 97) = .98, *p* = .373; Mental Rotation Accuracy, *F* (2, 97) = 1.66, *p* = .196; Average Maze Errors, *F* (2, 97) = .38, *p* = .686; Mental Rotation Time and Average Maze Completion Time Composite, *F* (2, 97) = .20, *p* = .823; Plus Minus Interference Score, *F* (2, 97) = .01, *p* = .995; Plus Minus Interference Time, *F* (2, 97) = 2.29, *p* = .107; Stroop Interference Accuracy, *F* (2, 97) = .35, *p* = .708; Eriksen Interference Accuracy, *F* (2, 97) = .03, *p* = .971; *F* (2, 97) = .98, *p* = .373; Stop It RT Go Trials, *F* (2, 97) = 2.03, *p* = .137; PASE score, *F* (2, 97) = .98, *p* = .378; PWB, *F* (2, 97) = .44, *p* = .648; PANAS Positive, *F* (2, 97) = 2.32, *p* = .104; PANAS Negative, *F* (2, 97) = 2.23, *p* = .113; SF-36 Mental Health, *F* (2, 97) = .75, *p* = .476; SF-36 Physical Health, *F* (2, 97) = 1.56, *p* = .215; SL, *F* (2, 97) = .57, *p* = .565.
