# Supplementary material for: The effect of cycling on cognitive function and well-being in older adults
Source: PLoS One. 2019 Feb 20;14(2):e0211779. doi: 10.1371/journal.pone.0211779 (PMC6388745; doi:10.1371/journal.pone.0211779)
Supplement: S3 File — Linear regression on the change scores. (DOCX) [file pone.0211779.s006.docx]

**Supporting Information 3 File**

When conducting a linear regression on the change scores for the different measures, there was no significant effect of total cycling time on any of the change scores for the measures that showed improvement after the cycling trial, *F*(1, 73) = 1.41, *p* = .240, *R*^2^ = 0.019 for StopIt Go RTs, *F*(1, 73) = 2.53, *p* = .116, *R*^2^ = 0.034 for Stroop IS, *F*(1, 73) = 0.95, *p* = .333, *R*^2^ = 0.013 for the SF36 Mental score.
