# Supplementary figures and images for: The effect of cycling on cognitive function and well-being in older adults
Source: PLoS One. 2019 Feb 20;14(2):e0211779. doi: 10.1371/journal.pone.0211779 (PMC6388745; doi:10.1371/journal.pone.0211779)

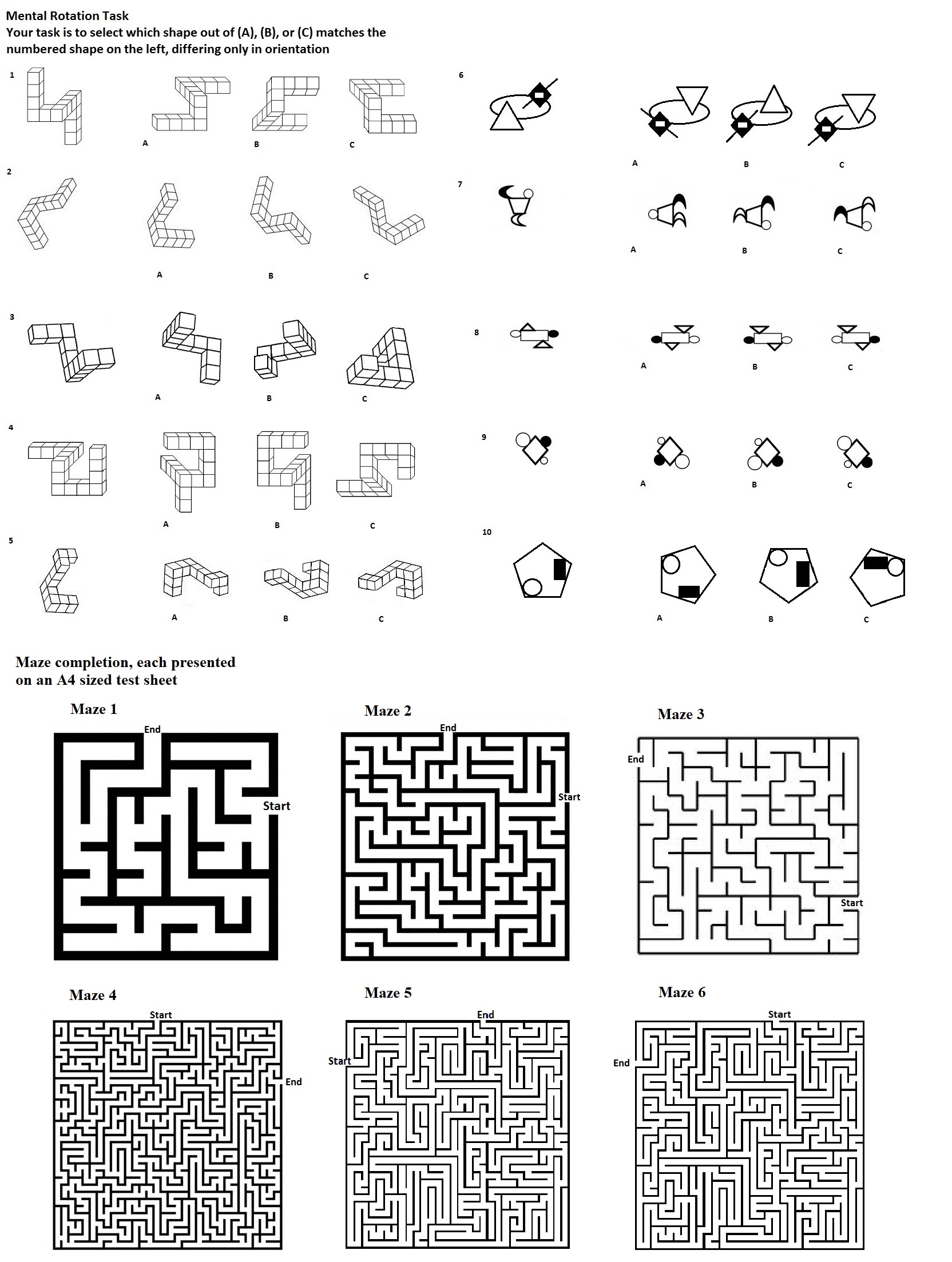

Supplement: S1 Fig — Stimuli used for the Mental Rotation Task and Maze Completion Task. (JPG) [file pone.0211779.s007.jpg]
